# A 53‐year‐old man with a 16‐year history of asymmetrical proximal muscle weakness, facial muscle weakness, and scapular winging

**DOI:** 10.1111/bpa.13171

**Published:** 2023-05-31

**Authors:** Jantima Tanboon, Rasha El Sherif, Michio Inoue, Mariko Okubo, Edoardo Malfatti, Ichizo Nishino

**Affiliations:** ^1^ Department of Neuromuscular Research National Institute of Neuroscience, National Center of Neurology and Psychiatry (NCNP) Tokyo Japan; ^2^ Departments of Genome Medicine Development Medical Genome Center, National Center of Neurology and Psychiatry (NCNP) Tokyo Japan; ^3^ Myo‐Care Neuromuscular Center, Myo‐Care National Foundation Cairo Egypt; ^4^ Université Paris Est Créteil, INSERM, U955 IMRB, APHP, Hôpital Henri‐Mondor Créteil France; ^5^ Department of Clinical Genome Analysis Medical Genome Center, National Center of Neurology and Psychiatry (NCNP) Tokyo Japan; ^6^ Present address: Department of Pathology, Faculty of Medicine, Siriraj Hospital Mahidol University Bangkok Thailand; ^7^ Present address: Neurology Department NewGiza University School of Medicine Cairo Egypt; ^8^ Present address: Department of Neurology Washington University School of Medicine St. Louis Missouri USA; ^9^ Present address: Center of Research in Myology Sorbonne Université Paris France

**Keywords:** asymmetrical weakness, muscle pathology, myopathy, PYROXD1, scapular winging

BOX 1 **Virtual glass slide**

Access at https://isn‐slidearchive.org/?col=ISN&fol=Archive&file=BPA‐22‐11‐289.svs

## 1 CASE PRESENTATION

A 53‐year‐old previously healthy man developed difficulty in climbing stairs and getting to stand at 37 years old. Carrying heavy objects and swallowing solids became difficult 5 and 11 years later, respectively. He became nonambulant at age 50 years after a surgery for bilateral hip fractures. There was no family history of neuromuscular disease or history of consanguinity. Physical examination showed predominantly proximal muscle weakness in the lower extremities, scoring 3 out of 5 by the Medical Research Council score. The upper limb weakness was mild and asymmetrical. There were bilateral scapular winging and facial muscle weakness, predominantly on the left side. He had nasal speech, decreased gag reflex, and palatal and tongue muscle weakness but no ophthalmoplegia or ptosis. Serum creatine kinase (CK) was 246 U/L (normal 22–171 U/L). Electromyography showed myopathic changes. Muscle MRI was not performed (Box 1).

## 2 FINDINGS

**FIGURE 1:**
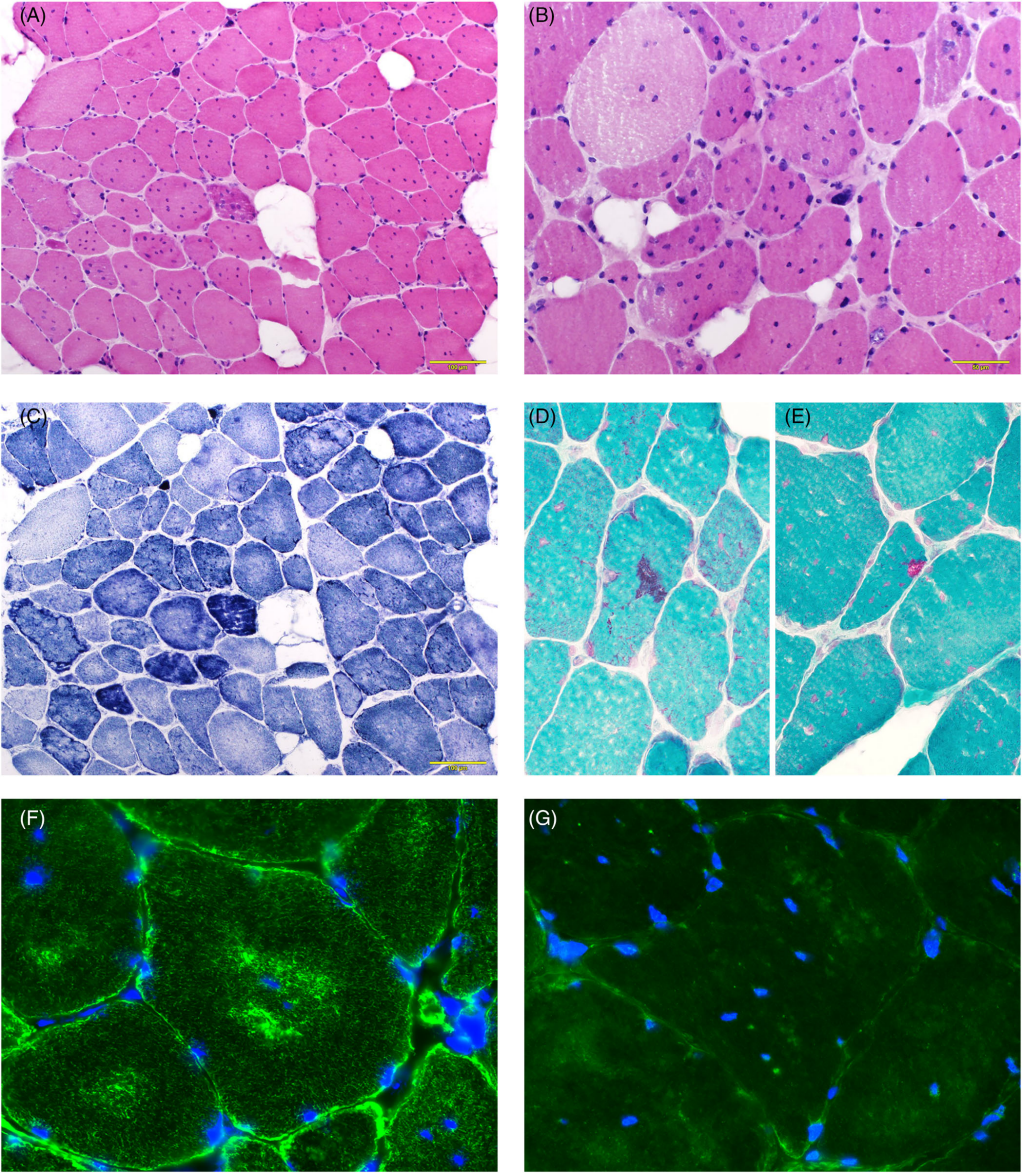
Marked fiber size variation with endomysial fibrosis and fat infiltration (A, H&E). Fibers with multiple internalized nuclei and pyknotic nuclear clumps (B, H&E). Disorganized myofibrils with core‐like appearance and lobulated fibers (C, NADH‐TR). Nemaline bodies (D, mGT) and rimmed vacuoles (E, mGT). Desmin and myotilin‐positive aggregates (F, G) (bars A, C = 100 μm; B, D, E = 50 μm; F, G = 20 μm).

**FIGURE 2:**
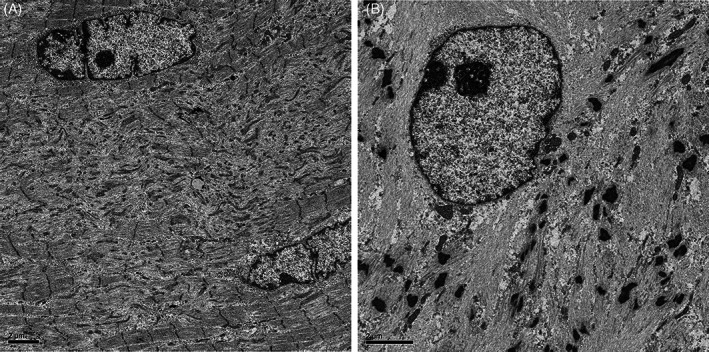
Streaming *Z*‐line (A). Enlarged nuclei and small fragments of enlarged *Z*‐line and electron dense materials resembling small nemaline bodies (B) (bars = 2 μm).

Hematoxylin and eosin (H&E) stain of a muscle biopsy from the left biceps brachii showed marked fiber size variation, scattered pyknotic nuclear clumps, fibers with multiple internalized nuclei (nuclear size 2.4–8.2 μm) (Figure 1A,B), and a few necrotic and regenerating fibers. There was mild endomysial fibrosis and mild endomysium and moderate perimysium fat infiltration. Nicotinamide adenine trinucleotide reductase (NADH‐TR) stain highlighted lobulated fibers and core‐like structures (Figure 1C). Modified Gömöri trichrome (mGT) stain revealed scattered fibers with nemaline bodies (Figure 1D), a few fibers with rimmed vacuoles (RVs) (Figure 1E), and some fibers with cytoplasmic bodies (not shown). Desmin‐ and myotilin‐aggregates were observed in some fibers (Figure 1F,G). Ultrastructural study showed streaming *Z*‐line (Figure 2A), enlarged nuclei and electron dense materials resembling small nemaline bodies (Figure 2B).

The main differential diagnosis for an adult‐onset, slowly progressive asymmetrical predominantly proximal muscle weakness with scapular winging, facial muscle weakness, and normal to slightly elevated CK level is facioscapulohumeral muscular dystrophy (FSHD). Asymmetrical weakness can also present in limb girdle muscular dystrophy, for example, LGMDR1 (*CAPN3*‐related) and LGMDR12 (*ANO5*‐related), and *FHL1*‐related X‐linked dominant scapuloperoneal myopathy (XSM). Notably, facial muscle weakness is less common in these entities. Histopathologically, FSHD, LGMDR1, and LGMDR12 can show dystrophic or inflammatory features. Lobulated fibers and eosinophils are reported in LGMDR1 while RVs are reported in LGMDR12. *FHL1*‐related XSM may contain RVs and desmin‐protein aggregates. Our case shares features described in *FHL1*‐related XSM and also contain myofibers with multiple internalized nuclei, nemaline bodies, and core‐like structures, raising the possibility of myofibrillar myopathy. Targeted next generation sequencing using our previously described “muscular dystrophy gene panel” based on the 2013 version of the gene table of monogenic neuromuscular disorders (Supporting Information), which also include *CAPN3*, *ANO5*, and *FHL1*, showed negative result. We identified a previously reported homozygous c.464A>G (p.Asn155Ser) variant in pyridine nucleotide‐disulfide oxidoreductase domain‐containing protein 1 (*PYROXD1*) gene on 12p12.1 [1] by whole exome sequencing and confirmed by Sanger sequencing.

## 3 DIAGNOSIS

Adult onset *PYROXD1*‐related myopathy.

## 4 DISCUSSION

PYROXD1 is a monomeric NAD(P)H‐oxidizing flavoenzyme in the nucleus and sarcoplasm of skeletal muscle that has co‐evolved with the RTCB catalytic subunit of the tRNA ligase complex (tRNA‐LC). It converts NAD(P)H at RTCB to NAD(P)^+^ to prevent oxidative inactivation of RTCB and to sustain tRNA‐LC activity in pre‐tRNA splicing and Xbp1‐mRNA splicing in unfolded protein response [2].

*PYROXD1* mutations are associated with early‐onset and adult‐onset myopathies; the age of onset ranges from neonate to late 50s. Interestingly, homozygous c.464A>G (p.Asn155Ser), the most common mutation, can present in both early‐ and late‐onset disease [1, 3]. The common presentations are symmetrical slowly progressive predominantly proximal, axial, bulbar, and facial muscle weakness. Restrictive pulmonary function, distal muscle weakness, scapular winging, high arched palate, joint hypermobility, and myopathic facies are noted. The less common findings include pes planus, pes cavus, pectus excavatum, joint contracture, scoliosis, spine rigidity, ptosis, calf hypertrophy, and cardiac involvement [1, 3]. Mild specific learning disabilities are reported in one childhood‐onset patient [1]. Asymmetrical weakness is reported in one adult‐onset patient. Most early‐onset patients are still ambulant in teenage years and adulthood although requiring supporting aids. A few neonatal‐onset patients who became non‐ambulant started using wheelchair in preteen while the childhood‐onset and adulthood‐onset patients started using wheelchair in adulthood and elderly adulthood, respectively. Most patients have normal to slightly elevated CK level. On MRI, gluteus maximus, quadriceps, sartorius, and gracilis are markedly affected while rectus femoris is relatively spared. Gastrocnemius is commonly affected in patients with homozygous c.464A>G (p.Asn155Ser). The common pathologic findings include fiber size variation, multiple internalized nuclei, endomysial, fibrosis, fat infiltration, and core‐like features. Myofibrillary changes including sarcoplasmic disorganization, nemaline bodies, and desmin‐, myotilin‐, alpha‐actin‐, and αB‐crystallin‐positive inclusion are observed [1, 3].

In conclusion, our patient demonstrates features described in *PYROXD1*‐related myopathy. Attentive clinicopathological correlation and including *PYROXD1*‐related myopathy in the differential diagnosis list and sequencing panel for myofibrillar myopathy would increase diagnostic yield for this rare entity.

## AUTHOR CONTRIBUTIONS

**Jantima Tanboon**: acquisition of data, interpretation of pathological results, drafting and revising manuscript for intellectual content. **Michio Inoue, Mariko Okubo, and Rasha El Sherif**: collecting clinical data and revising manuscript for intellectual content. **Edoardo Malfatti**: interpretation of pathological results and revising manuscript for intellectual content. **Ichizo Nishino**: design and conceptualize the study, acquisition of data, interpretation of pathological results, drafting and revising manuscript for intellectual content. All authors read and approved the final manuscript.

## CONFLICT OF INTEREST STATEMENT

The authors report no conflict of interests.

## ETHICS STATEMENT

All clinical information and materials used in this study were obtained for diagnostic purposes with written informed consent. This study was approved by the ethics committee of the National Center of Neurology and Psychiatry in Japan.

## Supporting information


**Data S1.** Supporting Information

## Data Availability

The data that support the findings of this study are available from the corresponding author upon reasonable request.
