# Supplementary material for: A 53‐year‐old man with a 16‐year history of asymmetrical proximal muscle weakness, facial muscle weakness, and scapular winging
Source: Brain Pathol. 2023 May 31;33(5):e13171. doi: 10.1111/bpa.13171 (PMC10467031; doi:10.1111/bpa.13171)
Supplement: Supplementary file 1 — Data S1. Supporting Information [file BPA-33-e13171-s001.docx]

**Supplementary file**

**List of genes in the targeted next generation sequencing “muscular dystrophy” gene panel^1^ based on the 2013 version of the gene table of monogenic neuromuscular disorders^2^.**

*AGRN, ALG13, ANO5, B3GALNT2, G3GNT1, CAPN3, CAV3, CHKB, COL12A1, COL6A1, COLGA2, COL6A3, DAG1, DES, DMD, DNAJB6, DOK7, DOLK, DPAGT1, DPM1, DPM2, DPM3, DYSF, EMD, FAT1, FHL1, FKRP, FKTN, FLNC, GFPT1, GMPPB, ISPD, ITGA7, KLHL9, LAMA2, LARGE, LMNA, MEGF10, MICU1, MYOT, PLEC, POMGNT1, POMGNT2, POMT1, POMT2, PTRF, SGCA, SGCB, SGCD, SGCG, POMK, SMCHD1, STIM1, SYNE1, SYNE2, TCAP, TMEM43, TMEM5, TNPO3, TRAPPC11, TRIM32*

**References**

1. Nishikawa A, Mitsuhashi S, Miyata N, Nishino I. Targeted massively parallel sequencing and histological assessment of skeletal muscles for the molecular diagnosis of inherited muscle disorders. J Med Genet. 2017;54:104-110.
2. Kaplan JC, Hamroun D. The 2013 version of the gene table of monogenic neuromuscular disorders (nuclear genome). Neuromuscul Disord. 2012;22:1108-35.
